# The Impact on Urology Residents’ Learning of Social Media and Web Technologies after the Pandemic: A Step Forward through the Sharing of Knowledge

**DOI:** 10.3390/healthcare11131844

**Published:** 2023-06-25

**Authors:** Severin Rodler, Cristina Eliza Bujoreanu, Loic Baekelandt, Gabriele Volpi, Stefano Puliatti, Karl-Friedrich Kowalewski, Ines Rivero Belenchon, Mark Taratkin, Juan Gomez Rivas, Alessandro Veccia, Pietro Piazza, Diego M. Carrion, Giovanni Enrico Cacciamani, Francesco Esperto, Enrico Checcucci

**Affiliations:** 1 Department of Urology, LMU University Hospital, LMU Munich, 81377 Munich, Germany; 2 Department of Urology, Medicover Hospital, 400489 Cluj-Napoca, Romania; 3 Department of Urology, University Hospitals Leuven, 3000 Leuven, Belgium; 4 Department of Surgery, Candiolo Cancer Institute, FPO-IRCCS, 10060 Turin, Italycheccu.e@hotmail.it (E.C.); 5 Department of Urology, University of Modena and Reggio Emilia, 42121 Modena, Italy; 6 Department of Urology, University Medical Center Mannheim, University of Heidelberg, 68167 Mannheim, Germany; 7 Urology and Nephrology Department, Virgen del Rocío University Hospital, Manuel Siurot s/n, 41013 Seville, Spain; 8 Institute for Urology and Reproductive Health, Sechenov University, 119435 Moscow, Russia; 9 Department of Urology, Hospital Clinico San Carlos, 28040 Madrid, Spain; 10 Urology Unit, Azienda Ospedaliera Universitaria Integrata Verona, 37126 Verona, Italy; 11 Division of Urology, IRCCS Azienda Ospedaliero-Universitaria di Bologna, 40138 Bologna, Italy; 12 Department of Urology, Torrejon University Hospital, 28850 Madrid, Spain; 13 Department of Urology, Universidad Francisco de Votoria, 28223 Madrid, Spain; 14 USC Institute of Urology, University of Southern California, Los Angeles, CA 90007, USA; 15 Department of Urology, Campus Biomedico, University of Rome, 00128 Rome, Italy

**Keywords:** surgical training, residents, urology, simulation, surgery

## Abstract

The COVID-19 pandemic has impacted urology residents and their training. However, several new technologies or knowledge platforms as social media (SoMe) and web-based learning solutions have filled this gap. Therefore, we aimed to analyze resident’s learning curves of new learning modalities, identify the evidence that is provided in the literature, and evaluate the possible impact of such solutions in the future. We conducted a non-systematic literature search using Medline, PubMed, and Embase. In addition, online resources of national and international urology resident societies were queried. The identified paper described SoMe, webinars, podcasts, pre-recorded surgical videos, educational platforms, and mobile apps in the field of urology that are used to gain access to information, teach and provide feedback to residents, and were used under the conditions of the pandemic. The application of those technologies harbors the risk of mis- and disinformation, but have the potential to provide access to education and validated knowledge, training, and feedback and thereby might democratize training of residents in urology globally.

## 1. Introduction

The coronavirus 2019 (COVID-19) pandemic has widely affected healthcare systems all over the world and, among its consequences, it has determined a slowdown of resident’s learning curve. Such consequences have been particularly relevant in the urological field [1,2,3]. In fact, in some countries, authorities have limited residents access to urology departments and suspended multiple surgical procedures in which residents are often involved in order to reduce infection transmission. Aiming to overcome the pandemic’s impact on residents’ learning curve, or at least mitigate it, various “alternative” teaching tools have been progressively introduced [4]. One of the most implemented instruments was represented by webinars, which allowed holding lessons and clinical case discussions, either by sharing multimedia content or by real-time interacting with experts. Another learning modality was represented by cloud platforms sharing pre-recorded lessons or surgical intervention, making them available for an on-demand consultation. This option is particularly suitable to show challenging cases commentated by experienced surgeons, explaining the “tips and tricks” of the procedure. Social media (SoMe) also offered some great opportunities regarding knowledge-sharing, such as Journal Clubs on Twitter, in which residents can discuss specific topics, having the chance to interact with key opinion leaders as well. A further teaching option was podcasts, consisting of pre-recorded audio files with lessons held by world-known opinion leaders.

Prior to the pandemic, urology residents had already started to use smart technologies such as SoMe for knowledge acquisition, for example [5]. During the pandemic, urology residents’ perception regarding these smart-learning modalities was evaluated with a survey that was completed by 501 participants from 58 different countries. The results were impressive, with 78.4%, 78.2%, 56.9%, and 51.9% who rated prerecorded videos, webinars, podcasts, and journal club on SoME as very useful learning tools [6].

Thanks to the huge success achieved by these alternative learning modalities, these tools are still implemented today in the post-pandemic period. Nowadays, the resident’s learning process can be considered as hybrid, with the new smart learning options supporting the traditional teaching modalities.

The aim of this review is to summarize the impact of new learning modalities on residents’ learning curves and to evaluate their possible future affect.

## 2. Materials and Methods

A non-systematic review of the available literature was performed. Medline, PubMed, and Embase were used as search engines for clinical trials, randomized controlled trials, review articles, and prospective and retrospective studies regarding the use of SoME, digital, and web technologies for urology residents’ learning.

We first defined three areas of interest and then determined research strings for search on the mentioned databases.

First, we evaluated the role of SoMe for residents’ learning (Section 3.1) with the research string: (1) (social media) AND (urology); (2) ((social media) AND (urology)) AND (learning).

In Section 3.2, we analyzed smart technologies for residents’ teaching with the research string: (1) urology AND teaching; (2) ((teaching) AND (resident)) AND (urology); (3) ((training) AND (resident)) AND (urology); (4) ((smart technology) AND (education)) AND (urology). 

Finally, in Section 3.3, the impact of medical/scholar apps for medical and surgical training was investigated following these criteria: (1) ((surgical training) AND (app)) AND (urology); (2) ((education) AND (app)) AND (urology) (3) ((training) AND (app)) AND (urology).

In addition, online resources of national and international urology resident societies were queried, and information was included in the manuscript.

## 3. Results

### 3.1. Social Media (SoMe) and Residents Learning

PubMed publications regarding SoMe and urology revealed the increasing online interest in learning about urologic disease (prevention, screening, treatment options) and even promoting healthcare services or funding them.

Telemedicine, which refers to remote clinical service and is under the umbrella of telehealth, had a promising start due to the COVID-19 pandemic context, but still has debatable aspects such as privacy and data protection, costs, and technical issues. Due to these limitations, it has not yet been made feasible in institutions and there is a lack of clear programs of implementation [7,8,9].

The need for accessible urological services/consultations will rise in the coming years as the elderly population is increasing. Therefore, investing in online technology is a smart choice, but not devoid of limitations, such as the loss of the conventional physical examination. Moreover, it would be an opportunity to lower the burden of unnecessary hospitalizations, disease severity, and progression through online patient education [10]. Crowdfunding campaigns were reported for urologic cancer care, impacting patients and their community, and lowering the financial burden of treatment. Testicular cancer initiatives were most successful and the number of SoMe shares was associated with financing [11]. One of the first validated urine biomarker assays for interstitial cystitis was developed using crowdsourcing by doctors [12].

Given the above context in which SoMe is continuously impacting both patients and doctors, the relationship between them is also changing. Urology residents must be trained to adapt and use technology safely and build a different relationship with their patients and mentors compared to other generations of urologists. Their spot in the professional field and their networking are also being influenced more and more by SoMe. A narrower look in the literature focused on learning ((SoMe) AND (urology)) AND (learning)) revealed less than 35 articles, out of which 44% were about the urology residency experience.

Even before pandemic times (2017), new media was reported important in urology education, with residents in Canada and Germany spending 45% of their education time on apps—54%, on SoMe—23%. A total of 90% of the surveyed residents watched online medical videos for education [13].

A study from 2019 showed the most used SoMe platforms in Urology by patients in the following order: Facebook, Instagram, Twitter, Snapchat, TikTok, with Facebook outreaching by far all of them [14]. Another surprisingly used SoMe platform in Urology is Pinterest, offering visual content. However, a study about urologic malignancies on Pinterest showed that 75% of the pins were of low or moderate quality with misinformative content in 4–26% of all genitourinary cancer pins [15].

COVID-19 flourished the perspective on online usage for medicine, putting it in the spotlight. Starting with residency application programs, the pandemic brought 1.315 virtual open houses through Twitter, Instagram, and Facebook in the USA [16]. Students applying to Urology residency increasingly used Twitter for learning, networking, and personal branding [17]. Twitter was reported as an important source of information for the virtual interview process [18]. Social media use had a significant impact in the AUAMATCH—American Urology Residency Match during the pandemic and it is probably going to continue as an important trainee recruitment in the future [19].

Twitter was also popular in creating an international urology journal club (#urojc) with a perspective in becoming an educational platform with global participation from consultant urologists and trainees [20]. Urologic knowledge acquisition is influenced by SoMe; in Saudi Arabia, urologists reported its moderate to high impact on their learning with YouTube being by far the most used source to watch and understand surgical skills [14]. Therefore, online education is becoming a powerful tool for urologists in international training through new digital content such as SoMe or podcasts, overcoming the reduced access to surgical procedures [21]. The COVID pandemic implied a reduced clinical and surgical volume with limited access to surgery, an aspect that is vital, especially for urologists in training who need to make the most out of their learning period that will define them as specialists. Hard times made them and their mentors resourceful and adaptable, developing a strong base for international telemedicine and telehealth. 

There is a need to continue developing smart learning and building safety around it, both for patientcare and teaching [16,17], as SoMe will be continuously transforming the patient–doctor and doctor–doctor interactions [22]. Moreover, SoMe is a smart tool for professional development and promotion, that could capitalize networking in research collaborations and increased citations. Based on our findings, we propose rules for beginners that use SoMe as part of their residency (Table 1). Social media is, and will be, present in our life, so it is smart to learn how to use it in our advantage, minimizing its risks [23]. There is a real danger of low quality or misleading medical information on SoMe and the medical community should address it. SoMe is a powerful tool in both ways and its use can also transform into a tragedy if a legal framework from authorities does not filter it, such as advertising fake new treatments to people that are desperate by their disease, resulting in treatment delay or even failure as death [20,21]. This applies to all specialties, not only Urology [24].

### 3.2. Smart Technologies for Residents’ Teaching

The increased use of smart technology has helped to mitigate the pandemic’s impact on urology residents’ training. These technologies include webinars, podcasts, mobile apps, and online educational platforms that have enabled residents to minimize the impact of the pandemic’s challenges (Table 2). These resources offer new opportunities for remote and interactive learning, enabling urology residents to engage with experts and peers, access expert insights and case discussions, and stay up to date with the latest research and clinical practices. In this section, we will explore the use of these smart technologies in urology residency training and their impact on resident learning.

Webinars provide interactive learning experiences that enable urology residents to engage with experts and peers, ask questions, and receive real-time feedback. Organizations have used this format to deliver educational content to their members. For example, the European School of Urology (ESU) has organized a series of webinars covering various aspects of urology, including surgical techniques, oncology, and pediatric urology. Similarly, the American Urological Association (AUA) has organized several webinars covering topics such as prostate cancer, bladder cancer, and male sexual dysfunction. From one of these web-based lectures, namely “Urology Collaborative Online Video Didactics Lecture Series” established by the University of California at San Francisco, Yi Li et al. [25]. found that case-based and guideline-based lectures were better rated or perceived than lectures around surgical techniques or updates in clinical practice [26,27]. This was also confirmed by Campi et al., where interactive webinars on clinical cases and recorded videos on the guidelines were preferred combinations, with 52.9% and 48.5%, respectively [6]. This could be explained by the fact that interactivity when discussing cases breaks the short attention span and passivity of participants, which are well-known difficulties of online teaching tactics [28]. Several questionnaire-based surveys confirmed the added value of webinars; for example, more than 50% of Dev et al.’s respondents were convinced that this technology should remain in current training [29]. Consequently, we see this through the trend of hybrid congresses, pushed by technological advances in software and organization in online broadcasting. Zeeshan Hameed et al. found that more than half of their responders preferred a hybrid congress as a format after the COVID-19 pandemic [30].

However, there are also some drawbacks, such as dependence on technology and less human touch or inter-individual interactivity and networking possibilities. To counter this disconnect, one should exploit variety and interaction by offering panel sessions, Q&A sessions, and live polls. This will ensure continued participation of residents in webinars and combat digital fatigue.

With recorded webinars and the free accessibility of these videos, this becomes an invaluable resource as they are kept online for future residents and students. A global web-based survey showed that it is mainly pre-recorded surgical videos that are preferred to watch [6]. This allows learning from them independently when you want and where you want. In a systematic review by Youssef et al., 14/22 studies (63.6%) showed a significant improvement in knowledge/skills after video-based teaching interventions, and in 13.6% they saw an improvement in trainee satisfaction [31]. Partly due to the pandemic, the supply of surgical step-by-step videos has increased tremendously, leading to a huge database that can be found on the various educational platforms, such as on the European Association of Urology (https://uroweb.org/, accessed on 21 June 2023), Surgery in Motion (https://www.europeanurology.com/surgery-in-motion, accessed on 21 June 2023), and AUA University (https://auau.auanet.org/content/surgical-video-library-2020, accessed on 21 June 2023).

Another modality that has gained popularity are podcasts, audio broadcasts that are distributed on the internet and accessed through different platforms such as websites, Spotify, iTunes, and so on. Podcasts tend to offer a more informal learning experience, making them a convenient option for residents who prefer to learn on the go. Compatible at any time and from any location, they are a flexible tool that residents can fit into their busy schedules. In the past, some major medical journals already produced podcasts, however, here too we see an exponential growth in supply and use since the pandemic [32]. Even so, Ptasznik et al. reported that 84% of educational urology podcasts on Spotify were created post-COVID-19 outbreak [33]. As such, many students testified that the medium is flexible and portable, allowing you to multitask and accomplish two goals at once [34].

With the global diffusion of technology, smartphones have become an integral part of not only our daily lives but also our work as medical professionals. A mini review by Mantica G. et al. identified 172 urological apps, finding that there were 41 apps on the market with education for and use by clinicians as a goal [35]. Another group were the practical tools, 21.5%, and these then provided a score calculator or medical values based on clinical data that are offered by the user. We see an exponential growth in the number of applications on marketplaces such as Google Play and the Apple App Store. For example, if we compare the study by Makanjuola et al. in 2012, we see eight times more applications with the same keyword search in 2020 [36]. The lack of scientific evaluation of applications is one of the drawbacks in their use; the current ratings of these are based on user feedback and thus are not evidence-based validated. Further in-depth coverage of these mobile applications for urology resident training as well as fields of application follows in Section 3.3.

Due to the successful implementation of these applications with webinar lecture series, hybrid conferences, education platforms, and the ongoing nature of the pandemic, it seems that this will not fly by and will be retained for the foreseeable future.

### 3.3. The Impact of Medical/Scholar Apps for Medical and Surgical Training

Medical and scholar apps emerged early after the introduction of smartphones and are a potential cornerstone of medical and surgical training. Already in 2015, 126 urology apps were identified by a study in Ireland, where they were already used by residents as an educational and reference tool in clinical practice [37].

In a first step, we identifyied medical education apps in the area of urology from the literature as described (Table 3). Only three publications were identified that described the use of apps for the training of residents. Keane et al. described the “Urology Med” app that is designed for medical students as an adjunct for traditional learning. The app includes knowledge on common urological conditions. As an interactive feature, the app provides five clinical cases and six quizzes as well as checklists for urology experiences during training [38]. In addition, the Society for Improving Medical Professional Learning (SIMPL) app has been identified through the literature search. This app supports the evaluation of surgical performance of residence and is rated as superior to conventional feedback systems [39]. The tool has previously been used to understand gender disparities and their impact on perceived operative autonomy and performance in a urology program [40].

In a second step, we analyzed the current landscape of apps in Germany as a large European country with multiple urology residency programs and a strategy for education apps by the national resident society (Table 4). Since 2014, the German Society of Residents in Urology (GeSRU) has developed app support for every step of residency [41]. The main app is designed as a logbook, where surgical procedures can be documented and then transferred to the state chamber as proof for becoming a specialist. Further, multiple surgical procedures are explained by videos and checklists are provided to support the daily life of a resident [42]. A training app for testicular cancers supports residents with correct staging and aftercare in this complex disease [43]. Further, an app for urological emergencies helps residents with validated knowledge to master the most frequent emergencies including renal colic, acute scrotum, and urinary retention.

Besides the apps provided by GeSRU, German residents have access to further commercially available or industry sponsored programs. This includes a non-urology-specific academic knowledge database covering all urologic diseases [44] and indication-specific apps focusing on, for example, prostate cancer [45].

The literature research as well as the example from the German resident landscape reveals the huge potential of medical training apps that can be potentially applied in the following areas of training or education:

(1) Education and validated knowledge:

Validated knowledge is crucial for residents as especially online it is difficult to distinguish reliable sources [46]. Further, knowledge has to be ubiquitously available and ideally accessible offline when necessary. Healthcare systems are further traditionally poorly equipped with infrastructure supporting healthcare professionals. However, smartphones are an ideal infrastructure [47] and, therefore, mobile apps might solve the above-mentioned issues.

Repetition is crucial for learning new topics [48]. Therefore, having the same library for a quick search when necessary during residency and for learning structured topics might speed up the time to acquire new knowledge. Traditionally, both things are separated. New topics are learned in lectures or in books. For online searches, search engines such as www.google.com have been established. However, when using search engines, residents might end up on different websites, each time reading new topics from various sources. When using the same app every time for searches and for learning, the repetition effect might apply quicker. In addition, residents use validated databases that way.

Motivation to acquire new knowledge might be crucial to succeed in residency. The combination of digital searches and knowledge databases can track the progress of residents and compare this progress to the progress of others. Apps can then generate scores and thereby increase motivation through gamification [49].

(2) Structured evaluation:

Feedback is crucial to develop skills during residency—especially in surgical fields [50]. Traditionally, feedback was given on a personal basis in an unstructured way and is influenced by many confounders [51]. Digital tools provide the opportunity to overcome those limitations, as already demonstrated by the before mentioned SIMPLE app [39]. Feedback can be actively requested via apps and especially superiors can be reminded by the system to provide feedback. In addition, residents can be reminded at predefined time points to request feedback (monthly, quarterly, yearly) which can be forgotten by a stressful daily routine. In addition to the quantity of feedback, apps can provide more structured feedback by tools that are suitable for the requested situation. Templates might guide feedback towards different dimensions of feedback that goes beyond technical feedback on performance and also includes personal skills [52].

(3) Curriculum/Organization

Resident programs are heterogenous but ultimately the same skills have to be acquired. However, many institutions do not offer structured programs in urology [53]. Therefore, apps and included checklists can be valuable tools to organize a residency. In addition, experiences and checklists can be shared between residents and thereby foster a high training quality through self-control of the progress. In addition, organizational criteria have to be met during residency. For example, depending on the country, residents have to prove a certain number of procedures to become specialists [54]. Those checklists can be included into smart curricula that residents have at hand at any time during residency.

Despite the great potential of apps in urology training, there are factors that limit the current applications. Currently, the development costs are higher than web-based solutions. In addition, yearly technical updates are required at least for iOS applications [55]. Keeping apps up to date and meeting regulatory hurdles will remain a major challenge for all training apps [56].

Interestingly, no criteria are developed for recommendation regarding the use of apps in medical training. Scientific evaluation of those solutions is highly limited and there is a lack of data evaluating the current tools. Conversely, the use of medical apps either for education of students or therapy of patients is not part of medical curricula. Therefore, a major step will be to increase knowledge of residents in digital technologies and how to leverage them in urology.

## 4. Discussion

Recently, our world has been heavily impacted by the COVID-19 pandemic and many aspects of our social and working life were revolutionized. The groundbreaking impact of this unprecedented event on healthcare systems has led healthcare professionals and politicians to deeply revise healthcare system functioning [57]. 

At the time of this manuscript writing, over 755 million confirmed cases of COVID-19 infections and over 6.8 million deaths have been reported globally. Furthermore, around the world, over 6.7 million new cases and over 64,000 deaths were reported in the last 28 days (16 January to 12 February 2023), a decrease of 92% and 47%, respectively, compared to the previous 28 days [58].

Concerning the European Region, over 1.2 million new cases were reported, with a decrease of 52% compared to the previous 28 days. Six (10%) of the 61 countries for which data are available reported an increase in new cases of 20% or greater. It means that the pandemic is continuously evolving since 2020.

Therefore, we can define three different phases of the pandemic during which, in each of them, surgery, patients’ care, and education have progressively changed.

The first one, in 2020, consisted of the “outbreak phase” during which, for the first time, physicians had to face the emergency trying to reshape patients’ management [1], and the surgical activity was heavily affected, limiting it to newly-defined emergency procedures [57,59]. Parallelly, new pathways of pre-, intra-, and post-operative care for urological patients undergoing urgent procedures or non-deferrable oncological interventions were established [60,61]. Focusing on residents/young urologists’ education, an overall decrease in daily residents’ exposure to all training activities from both clinical and surgical perspectives was registered, and this was even more pronounced for residents attending the final year of training [3]. With the aim to overcome these limitations, determined by the limited access to hospital facilities for residents, new technological tools emerged with an unprecedent speed. Webinars, journals’ club via social media, podcasts and pre-recorded surgical videos available on online cloud platforms became the new teaching methods for both theoretical and practical tasks [4]. A global survey with more than 500 answers from residents of different countries reported a good appreciation for these digital teaching modalities, especially for updating guidelines and surgical video sharing [6].

The second phase of the pandemic in 2021, was the “vaccine phase” characterized by the reorganization of outpatient and inpatient urological activities and a progressive implementation of telemedicine [10]. In fact, vaccination determined a restart of educational activities, such as training, congresses, and courses. Furthermore, the possibility to track vaccinated patients allowed the increase in urological activities, decreasing waiting lists. As reported in our Results section, telemedicine and telehealth could be the safest way to deliver urological care for a large percentage of patients [11], even if the main challenges for a real transition to telemedicine in the daily clinical practice are represented by healthcare digital infrastructures that remain inadequate, and the low access of patients to computers. In fact, health practitioners should become increasingly familiar with telemedicine and initiatives to improve patients’ digital “literacy” should be implemented [12]. Concerning educational aspects, the advent of new smart teaching modalities allowed to reduce the impact of COVID-19 restrictions; however, even if less burdensome than expected, urology residency training (especially in endoscopic surgery) was highly affected throughout the whole year [13]. Obviously, congress and meetings were affected by the pandemic, and the continuing medical education had to adapt [14]. Notwithstanding the quick and widespread diffusion of these so-called “webinar” meetings, the impossibility to virtually recreate human contact, affections, and emotions poses issues about the popularity of such entirely virtual formats. Following this concept, the idea of “hybrid” events, in which the potential of the digital world is integrated into the real world has been planned. In this format, the faculty is present on-site while attendees can enjoy the meeting on-line in a “phygital” dimension [14].

Finally, the third phase that characterized 2022 was the “variant phase” in which COVID-19 variants, such as Omicron, appeared. At this stage, healthcare systems were already prepared to face COVID-19 infections. Surgical and ambulatory activity restarted thanks the benefit of the vaccination campaigns, with the issue to dispose of waiting lists’ overload created during the most critical phases of the pandemic. Residents’ education could return to standard modalities with direct access to the hospital facilities. However, the lesson learned from the pandemic must not be forgotten. Now we have in our hands new technological instruments that can be used for both theoretical and practical training. SoMe and smart technologies should be integrated with the more traditional training modalities, allowing to continue the education process, sharing knowledge across the world, equalizing and democratizing the training opportunities for the urologists of the future.

Our review reveals the feasibility of online approaches for knowledge learning, as a quick adoption to the circumstances was required of the pandemic. However, there is still limited knowledge on the precise impact of online learning on the performance of urologists after their training and how urologists trained during the pandemic compare to urologists trained in a traditional way. It will be of high interest whether the addition of digital tools into conventional curricula can increase not only knowledge but also hands on lab or surgical skills.

The current study has not focused on virtual and augmented reality learning approaches as we focused on the impact of social media and web technologies on residents’ learning for this review. The two mentioned technologies might play a pivotal role in the training of especially practical skills. Simulators, for example, for robot-assisted radical prostatectomy, have revealed improved surgical outcomes when used during training [62]. As current technology for simulation matures, new solutions might be introduced to the curricula of residents.

This review is limited by its non-systematic design. There is the risk of overlooking key manuscripts in the field. However, experts in the field of resident teaching in urology either working at or with close contact to academic centers conducted this literature search and analyzed the results.

Tech-literacy will be a key skill of future residents to succeed in their careers. However, application of smart technologies including SoME warrants the awareness for mis- and false-information as well as new ways of teaching on how to leverage those technologies best during urologic training. Further, prospective data to evaluate those solutions and the impact on residents training beyond the pandemic have to be acquired as most solutions currently lack independent evaluation.

## 5. Conclusions

Residents training in urology has been dramatically impacted by the COVID-19 pandemic. New technologies including SoMe, digital teaching formats, and training apps have entered the curricula and impact residents’ lives prior and during the pandemic. Balancing risks as dis- and misinformation with opportunities such as increased access to education, training, and feedback remain a challenge beyond the current requirements of the pandemic. Sharing knowledge, democratizing education, and scaling skills of residents by the means of new technologies and platforms is a vision for the future and might benefit patients.

## Figures and Tables

**Table 1:** Social media (SoMe) decalogue.

Facebook is a platform that reaches a very high number of people, along with Instagram. Twitter is used highly by Urologists. YouTube is of high interest for surgery learning. Crowdfunding can be an option for your research idea. Choose a platform that suits you to create your voice/brand. This depends also on the online activity in each country—research about it!
2. Connect to like-minded peers for inspiration and be present in the online world to understand the background of information your patients have read before entering in your consultation room. It enables you to quickly address concerns and build a better communication in your daily practice.
3. Use SoMe to build trust and connection from online activity before the patient actually meets you face to face in the consultation room. This will result in good compliance and follow-up.
4. Focus on prevention/screening and informing patients—general Q&A in daily practice.
5. Do not use SoMe for consultations, there must be a legal online framework for it. Think about data protection and privacy! Get involved in programs created by institutions to enable online professional activity—it will flourish in the following years.
6. Do not mix personal and professional accounts.
7. Use SoMe to learn and find career opportunities. Is all out there, just pay attention to what you are searching/ scrolling!
8. Target online professional activity on networking for international collaborations.
9. Think twice before posting—once is out there, it remains.
10. Your online activity is your business card. Own it and use it!

**Table 2:** Smart technologies with their attributes and examples for each type.

Smart Technology	Key Message	Examples
Webinars	Provide interactive learning experiences that enable engagement with experts and peers, ability to ask questions and to receive real-time feedback	European Association of Urology (EAU) American Urological Association (AUA) Canadian Urological Association (CUA) British Association of Urological Surgeons (BAUS)
Podcasts	Offer convenient and accessible learning resources with possibility to learn on-the-go and access expert insights, case discussions, and updates on the latest studies	Urology Care Podcast The Modern Urologist AUA University AUA Inside Tract BJUI—BJU International EAU Podcasts
Pre-recorded Surgical Videos	Improve surgical technique and decision-making by detailed visualizations of surgical procedures, tips, and tricks from experts	Websurg European school of Urology (ESU) Surgery in Motion
Mobile Apps	Access to educational resources and tools on-the-go, such as surgical videos, risk calculators, case studies, and guidelines	eUROGEN UrologyMatch Mobile EAU Guidelines app AUA Guidelines app EAU NMIBC Risk Calculator
Educational Platforms	Comprehensive and flexible learning experience by offering access to a wide range of educational resources, online courses, and assessments	EAU e-learning Urology Match Medscape Urology

**Table 3:** Medical education apps in the literature (urology).

Name	Country	Content	Evidence
Urology Med [38] Version: 2023.3.22	Ireland	Urology reading material Clinical cases Self-assessment Checklist for procedures/activities	Download numbers App store rating
Society for Improving Medical Professional Learning (SIMPL) app [39,40] Version: 2.5.3	USA	Feedback System for urologic surgery via App	Survey during implementation

**Table 4:** Education apps from the German Society of Residents in Urology.

Name	Content
GeSRu App Version 2.1	Logbook for surgical procedures, instruction videos
GeSRu Hodentumor App Version 1.0	Classification, staging recommendation, and aftercare for testicular cancer
GeSRu Uro Emergency Version 1.0	Knowledge database for urological emergencies

## Data Availability

Data is contained within the article.
